# Dry aging of beef; Review

**DOI:** 10.1186/s40781-016-0101-9

**Published:** 2016-05-19

**Authors:** Dashmaa Dashdorj, Vinay Kumar Tripathi, Soohyun Cho, Younghoon Kim, Inho Hwang

**Affiliations:** Department of Animal Science and Biotechnology, Chonbuk National University, Jeonju, 561-756 Republic of Korea; Department of Livestock Production, Mongolian University of Life Sciences, Ulaanbaatar, 17026 Mongolia; Animal Products Research and Development Division, National Institute of Animal Science, RDA, Wanju, Republic of Korea

**Keywords:** Dry aging, Beef, Dry aging parameters

## Abstract

The present review has mainly focused on the specific parameters including aging (aging days, temperature, relative humidity, and air flow), eating quality (flavor, tenderness and juiciness), microbiological quality and economic (shrinkage, retail yields and cost) involved beef dry aging process. Dry aging is the process where beef carcasses or primal cuts are hanged and aged for 28 to 55 d under controlling environment conditions in a refrigerated room with 0° to 4 °C and with relative humidity of 75 to 80 %. However there are various opinions on dry aging procedures and purveyors of such products are passionate about their programs. Recently, there has been an increased interest in dry aging process by a wider array of purveyors and retailers in the many countries. Dry aging process is very costly because of high aging shrinkage (6 to15 %), trims loss (3 to 24 %), risk of contamination and the requirement of highest grades meat with. The packaging in highly moisture-permeable bag may positively impact on safety, quality and shelf stability of dry aged beef. The key effect of dry aging is the concentration of the flavor that can only be described as “dry-aged beef”. But the contribution of flavor compounds of proteolysis and lipolysis to the cooked dry aged beef flavor is not fully known. Also there are limited scientific studies of aging parameters on the quality and palatability of dry aged beef.

## Background

For centuries, dry aging was a common way for butchers to preserve and tenderize beef. Up to 50 years ago, dry aged beef was the norm, then with the advent of vacuum packaging along with increased efficiencies in beef processing and transportation, lost the dry aging process [1]. Thus there were small numbers of meat purveyors who actually participated in this type of aging process. However, recently there has been an increased interest in dry aging process by a wider array of purveyors and retailers in the United States and Australia [2]. Although there appears to be strong interest in Asian countries in dry aging, especially high end restaurants in many countries such as Korea, Japan, Singapore, Taiwan and Hong Kong are beginning to feature dry-aged beef on their menus. As demand for dry-aged beef increases, it created a high end niche in the food service market in Korea [3].

**Fig. 1:**
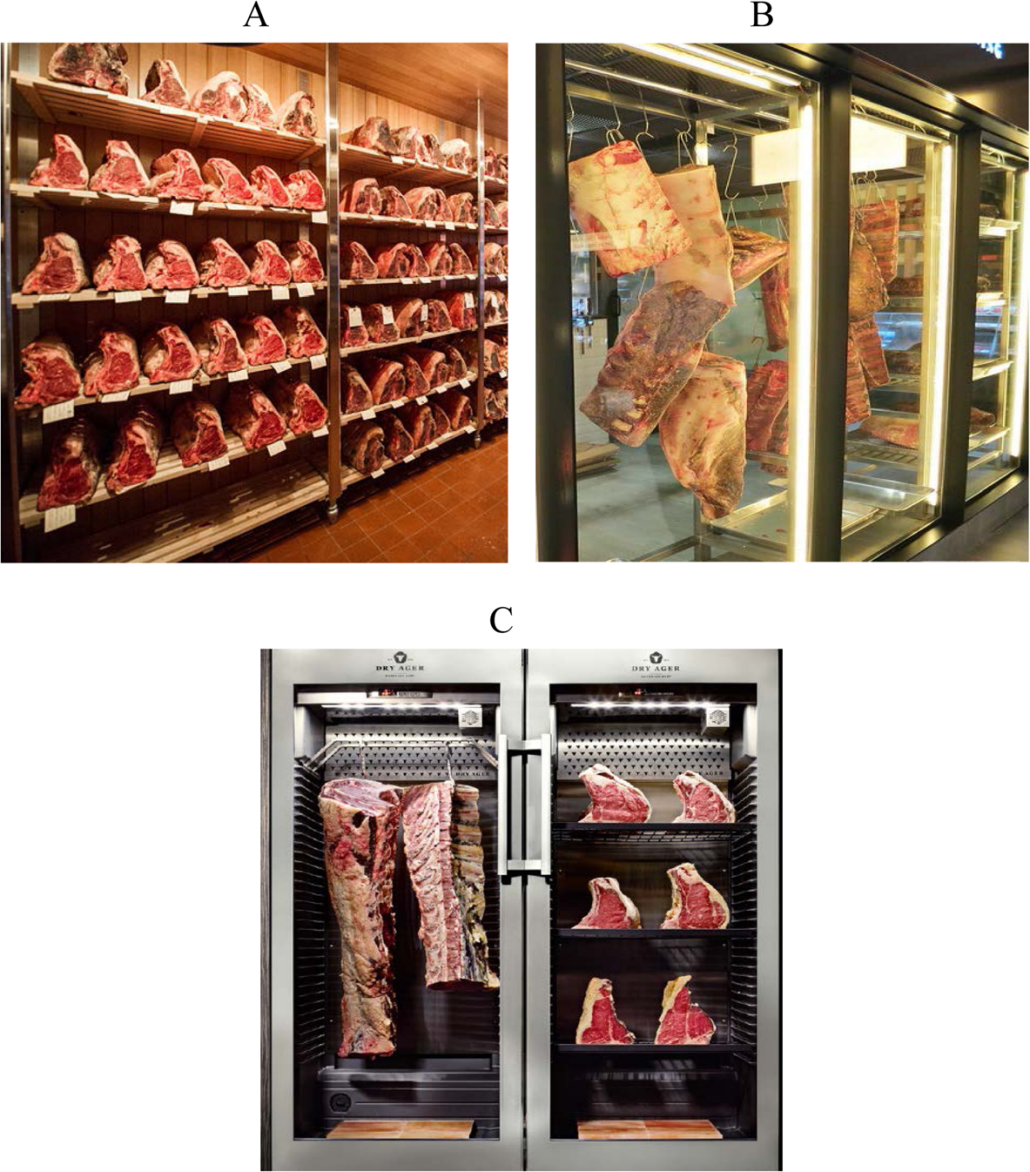
The refrigenated coolers for dry beef; **a** Typical dry aging room; **b** dry aging maturing display; **c** meat maturing fridge

In general, there are two forms of beef aging techniques: wet and dry which result in flavor development and more tender meat [4–7]. When beef is wet aged, it is put in a vacuum sealed package and stored in a controlled environment for a specific period of time. Dry aging is the process of hanging beef carcasses, subprimals or placing unpackaged primal cuts in a refrigerated room (Fig. 1) and left to age for several weeks or even months at controlled temperature, relative humidity and air flow [8, 9].

The key effect of dry aging is to concentrate the flavor that can only be described as “dry-aged beef” [1, 4, 6]. During the dry aging process, the juices are absorbed into the meat, chemical breakdown of protein and fat constituents occurs which result more intense nutty and beefy flavor. Moreover, during aging the beef’s natural enzymes break down the proteins and connective tissue in the muscle which leads to more tender beef [10].

Furthermore, dry aging process is costly relative to other conventional processing methods, because of high aging shrinkage, trim loss, risk of contamination, and requirements of aging conditions and space. It is a very time consuming process and needs special care along with a large and evenly distributed fat content in meat. Therefore, only the highest grades of beef with necessary marbling can be dry aged. The main reason behind that dry aging is not universally done anymore because it takes additional costs for processors [11–13]. On the contrary, there exists a small niche market of consumers who prefer and are willing to pay for the unique flavor of dry aged beef. Dry aged steak is offered in mostly fine restaurants, upscale grocery stores and gourmet steak companies due to the taste is almost incomparable to that of wet aged or vacuum-packaged.

To the best of the authors’ knowledge limited scientific studies have evaluated the role of the aging parameters on the quality, palatability, and shrinkage of dry-aged beef. The aim of this review is to discuss the specific parameters including aging, eating quality, microbiology and economic involved in beef dry aging process, which may be useful to companies or retailers who are interested in producing and marketing dry-aged beef.

## Fabrication subprimals and steaks for dry aged beef

There are various opinions on dry aging procedures and purveyors of such products are passionate about their programs. The most purveyors dry-aged beef carcasses or primal cuts at least 21 days or longer depending on desired flavor profile. Generally after the animal is slaughtered and cleaned, the carcass is halved and either the 2 sides are hung in a cold room at 2 °C for 21 d. Then, after 21 d each side is divided into the primal cuts (eg. chuck, loin and rib). The primal cuts (except loin and rib) are then cut into roasts (eg. topside, silverside, brisket), or cut for stewing beef or minced. After hung or place the loin and rib for a further 7 d and at 28 d, cut into rib-roasts and steaks (eg. fillet, sirloin, T-bone). The steaks are then packaged and are ready for sale.

### Fat content and beef grade

The dry aging process typically requires beef with ample marbling to help to ensure and finished products with consistent flavor and juiciness. Carcasses with Modest or Moderate marbling, representing the upper two-thirds US Choice grade commonly referred to as Top Choice in the US beef industry, and carcasses that represented the entire range of Slight marbling for the US Select grade [14]. Dry aging is commonly done on products that are of higher quality grades, upper two-thirds USDA Choice and USDA Prime, whereas limiting loin selection to marbling levels to low Choice and Select within the “A” maturity [15]. The significant higher rates were in USDA Choice ribeye steaks for juiciness, overall palatability and overall like than USDA Select ribeye steaks [16–18].

Premium dry aged beef products usually come from grain fed cattle due to the greater marbling within the meat. Marbling pattern required for successful dry aging means that only higher graded beef can be dry aged. Marbling adds flavor and is one of the main criteria for judging the quality of cuts of meat. When marbled steaks are grilled, the fat specs melt into the meat and make them tender and juicy, with a distinctive buttery flavor. Furthermore, number of studies demonstrated that the effect of fat on tenderness related to connective tissues of aged beef: The loin’s adipose tissue deposits between the muscle fiber bundles appeared to partially disrupt the honeycomb structure of the endomysium, the perimysium separated into thinner collagen fibers [19].

### The most typical subprimals and steaks for dry aged beef

The most typical dry-aged paired beef subprimals as a loin (short and strip loins), top sirloin butts, beef ribs (short ribs and ribeye roll) are used to produce typical steaks. The tenderest and most expensive cut of beef is the tenderloin (also known as fillet roast, fillet steak, and fillet mignon) is a muscle in the loin primal and is in two different subprimals, the short loin and sirloin, creating its unique oblong shape. The porterhouse steak is from the portion of the short loin that is closest to the sirloin section, as having tenderloin that measures at least 1.25 inches in width parallel to the backbone of the steak (the “T” portion of the bone). The T-bone steak is what the rest of the steaks from the short loin are called. This steak is highly prized in leading steakhouses because this porterhouse cut is comprised of the largest portions of a tenderloin and New York steak [20].

If the tenderloin is removed and sold separately as fillet roast and fillet steak, the remaining piece is sold as a strip steak. The strip loin bone is the long narrow piece on a T-bone or porterhouse steak. This steak generally has a fair amount of marbling (tiny flecks of fat interlaced in the muscle) which gives the cut a good flavor profile and tenderness. This cut has many aliases, New York steak, Kansas City steak, Boneless Club steak or Ambassador steaks to name a few. The sirloin transverses the hip, so at one end is very close to a high priced porterhouse. The boneless steaks may be prepared from any top sirloin butt [20].

Rib steaks may be cut from any rib in the primal cut, with or without a bone. The cut, bone-in ribeye is found in the primal section known as the rib. The rib primal is located from rib six through twelve right behind the chuck section. This cut is one of the most well-known cuts, primarily from the popularity of the bone-in rib roast, also known as prime rib. This section of the animal is known for its marbling, flavor and tenderness. The bone-in ribeye steak is also referred to as, Ribeye steak, Cowboy steak, Spencer steak, Prime Rib steak or Saratoga Steak [20].

Thus, dry aging is usually done with primals on the bone. The primals from carcasses with “A” maturity and with the modest or higher marbling preferred for dry aging.

## Dry aging parameters

The primary factors to consider when developing dry aging guidelines include days of aging, storage temperature, relative humidity and air flow. All these factors must be closely observed and aligned in order to achieve a superior product with optimum tenderness and flavor concentration.

### The days of aging

There are various opinions on length of dry aging and purveyors of such products are passionate about their programs. Numerous researchers have reported that the most frequent range for dry aged subprimals is between 14 and 40 d, these days have all appeared effective in producing the desired results of this process [18]. Lepper-Blilie et al. [15] reported that the majority of the product being aged for 21 d. It appears that aging for 28 d does not significantly increase the unique dry aged flavor components compared with aging for 21 d [21]. While Smith et al. [17] reported that steaks aged for 21 d received the highest value for level of beef flavor compared to all other aging periods. However, any period beyond 21 d resulted in similar level of beef flavor ratings as compared to 14 d aging treatments.

Processors have found that the minimum amount of time to dry age beef that obtain good results is 28 d. The USMEF [22] also suggests that aging time range for dry aging from 14 to 70 d, while preferred range from 28 to 55 d could be acceptable. According to Perry [12], the aging process should be established between 50 and 80 d. There was a deeper and more complex flavor ribs of grass fed 36 month old Black Angus beef and Wagyu were firstly stored for 45 and 50 d. It was also reported that a 120 d dry aging process had not increased the flavor of beef at the same levels as they had observed between 35 and 80 d. Lately, there has been a fantastic arms race among chefs in search of new flavors through longer aging times: 35, 42, 56, 75 and more days. The “Saison” in San Francisco regularly takes its beef out to 90 d; “Pat LaFrieda” in New Jersey 120 d, Eleven Madison Park, 140 d; and, Mario Batali’s Carneino in Las Vegas features steaks so old 180–240 d [23]. However, extended dry aging over 100 days is an extremely personal preference.

The rate of aging is also temperature dependent. Meat Industry Services Australia [2, 24] reported a period of about 4 weeks at −0.5 °C would be required to achieve the same level of tenderness as 2 weeks at 5 °C. Whichever temperature is selected, the rate of improvement in tenderness is the highest during early stages of aging, and decreases with time.

### Temperature

Dry aging literature has mainly reported the optimum temperature is between 0° and 4 °C (32–39.2 °F) because storage temperature for dry aged beef should not differ from those for wet-aged beef products [1, 4–6, 8, 12, 16, 17, 25]. Aging temperature is critical to dry aging because if the temperature of storage is elevated, the enzymatic processes involved in aging will work quite well and improve palatability. However, higher temperatures also promote more rapid bacterial growth, resulting in the development of off-odors so aging is usually done at a temperature as low as possible without freezing the meat [1, 2]. Some other processors have recommended that if it is below the freezing temperature for meat the enzymatic processes involved in aging will slow, therefore, the ideal temperature for long-term aging is −0.5 °C ± 1 °C. If the product is aged for only 1 to 2 weeks, higher temperatures of 2 to 3 °C might be acceptable [2, 12].

Temperature stability is important. Meat Industry Services Australia [2, 24] recommended the dry aging room should have an ante room or open to another refrigerated area to prevent ingress of warm, moist outside air. The provision of a plastic-strip door will reduce entry of outside air when the door is open [2, 24].

### Relative humidity (RH)

Controlled relative humidity of the air plays a crucial role in the dry aging process because if the humidity is too high, spoilage bacteria can grow resulting in off-flavors. Although meat can sweat, creating an unpleasant sticky surface. If the humidity is too low will restrict bacterial growth, but promote greater evaporative weight loss and beef will dry out too quickly and therefore causes the steak to have less juiciness than is needed [12]. A relative humidity of 61 % to 85 % is recommended and actual RH should be recorded daily for the duration of the aging process [24; 21]. There are limited published studies that have compared the effects of different RH levels on dry aged beef. The studies in this area have used a RH of approximately 80 % [8, 12, 16, 17] and Campbell et al. [6] dry-aged beef in a cooler with 75 % RH and Warren and Kastner [4] used a range humidity of 78 ± 3 %.

### Air flow

There should be sufficient air flow to provide air circulation without dead spots or sites of high velocity. If not enough air the meat cannot release the necessary moisture to achieve the drying process, while if too much air, the meat will dry out too quickly and increases trimming losses in the final product [1]. The USMEF [22] recommended that an air flow range of 0.5–2 m/s (1.6–6.6 ft/s) for dry aging and a velocity of 0.2 to 1.6 m/s over the product should be sufficient. The air velocity and flow should be kept uniformly for the duration of the drying process, and it is most critical at the start of the dry aging process. The airflow can be controlled with a properly designed refrigeration unit, wire racks with stainless steel, perforated shelves, trees or hooks, supplementary fans, air filtration systems and ultraviolet light [10, 24].

Increasing the airflow around the aging room is needed to make sure that the fresh beef dries as quickly as possible. It is possible by using a number of ceiling mounted fans to push air in different directions around the room [12]. To prevent spoilage, portions of meat must be adequately separated from each other to allow efficient and controlled air flow between each portion [22]. The primal cuts to be dry aged should be placed fat side down on the shelves, so that the air can circulate around all sides of each cut. In the case of bone in cuts such as short loins, the cut should rest on the chine bone [2].

Considering these results, purveyors suggests that the parameters such as aging days 28 to 55, temperature 0 °C to 4 °C, RH 75 to 80 % and air flow 0.5 to 2 m/s are advisable for dry aging beef since it inhibits microbiological contamination, improves tenderization by aging and meat are more tasty. Thus, there are no scientific studies have evaluated the effect of different storage temperatures, relative humidity and air velocity on the quality and palatability of dry aged beef and limited results on aging periods.

## Microbiology/packaging

### Microbiology

Dry aging involves restricting bacterial growth and encourages the growth of beneficial mold. During the entire process of dry aging beef, molds from the *Thamnidium* are found on the surface of the meat. *Thamnidium,* which is the most desirable, appears as pale gray patches called ‘whiskers” on the fatty parts of aged beef. These organisms are important because their enzymes are able to penetrate into the meat. In fact, *Thamnidium* releases proteases and create collagenolytic enzymes which break down the muscle and connective tissues. As a result, these actions bring about tenderness and taste in the dry aged beef [24].

The growth of *Thamnidium* mould can start from 3 weeks after that the aging process has started [24]. *Rhizopus* and *Mucor genera* are other molds associated with dry-aged beef; however, they have been associated with human infectious diseases and do not provide any favorable characteristics for aging meat [24].

Dry aged meat products must be tested for mold to validate the procedure. Testing involves removing a 100 g portion of untrimmed aged meat that includes visible mold if it is present, and sending it for laboratory. If testing for mold shows that the results are positive and then confirmation that the mold is *Thamnidium* must be conducted [24].

With the proper handling practices, subprimals can be dry aged up to 35 days without any negative effect on flavor and safety. According to Campbell et al. [6], dry aged steaks had higher aerobic plate counts compared to controls; however duration of dry aging did not affect aerobic counts. This lack of response to dry aging time may have been because of growth inhibition caused by surface drying and storage temperatures low enough to retard growth. In addition dry aging relies on reduction of water activity on the surface to minimize bacterial growth. [2]. Dry aged samples aged 14 and 35d had lower (*P* < 0.05) water activity than wet aged 35d samples, because water activity decreases by physically removing water during drying [17].

If you do not plan to utilize the dry aged product immediately, do not fabricate the subprimals and keep them in the cooler. Dry aged meat would be trimmed just before sale, because trimmed and packaged dry aged meat cuts have generally a shelf life of 2 to 3 d. To confirm the wholesomeness of both dry and wet aged products, the shelf life must be validated by testing for *Enterobacteriaceae* and *E.coli*. The critical limits for wholesomeness for these purposes are microbiological limits of *Enterobacteriaceae* of 1,000 cfu/g and *E.coli* of 10 cfu/g [24].

Relative to this, University of Wisconsin Center for Meat Process Validation, [26] reported that generic *E.coli*, *coliforms* and *Enterobacteriaceae* were detected on 69 % (3.7 cfu/cm^2^), 84 % (5.8 cfu/cm^2^) and 93 % (7.3 cfu/cm^2^) respectively, of beef carcasses sampled before dry aging. But generic *E. coli*, *coliforms* and *Enterobacteriaceae* were only detected on 8 % (0.17 cfu/cm^2^), 17 % (0.23 cfu/cm^2^) and 37 % (4.9 cfu/cm^2^) respectively, of 6 days dry aged carcasses sampled. Thus, dry aging may be an effective intervention treatment against *E. coli*.

The critical limits for a dry aging step would be related to temperature, aging time, and relative humidity or air flow. Therefore antibacterial strategies such as ultraviolet (UV) lighting and air filtration systems, have also been employed. Air can also circulate through UV lit chambers however the costs may be prohibitive. The normal fluorescent lighting should be switched off in the room when it is not required [24; 12].

### Packaging/dry aging bag

The most beef sold in food stores are wet aged or vacuum packaged after 7 to 21 days of post mortem. Only a very small amount of meat is dry aged (no protective packaging), usually for 14 to 35 days [9]. Recently a relatively new kind of bag technology that has a highly water vapor transmission rate (TUBLIN® 10, TUB-EX ApS, Denmark) was introduced to the market. The TUBLIN® 10 bags are sold in the USA under the name “UMAI dry bag steak” by the company of the same name [8, 9, 21]. Dry aging in a this bag will produce dry aged flavor equal to that achieved with traditional dry aging. Researchers noted that the material in the bag functions as a breathable plastic and designed to decrease weight, trim loss and/or microbial contamination, and to increase yield, but to result in similar tenderness and other sensory traits as dry aged beef [8, 11, 21].

Li et al. [11] found that meat aged in dry aging bag was more tender and juicier and overall preferred by consumers compared with samples aged in vacuum. No differences were found in pH, smell, shear force, color, *Enterobacteriaceae,* and mold counts.

Furthermore, Ahnström et al. [8] compared dry aging of Angus beef loins for 14 and 21 days in bag with traditional dry aging methods. Results showed significant cut weight conservation and decreased trim with the bag-aged treatment. There were no differences in flavor, pH, moisture, fat, cooking loss, shear force and total plate counts (final 4.7 log cfu/cm^2^) between aging methods. Adipose tissue aged in the bag had more LAB (6.6 and 4.6 log cfu/cm^2^) than those dry aged (3.3 and 2.4 log cfu/cm^2^) after both aging periods. Yeast counts on lean tissue in the bags were lower (2.4 to 4.2 log cfu/cm^2^) than dry aged (4.2 and 5.2 log cfu/cm^2^) for both aging days. Mold counts for both tissue types among both treatments were less than 0.3 log cfu/cm^2^ during aging [8].

In addition, DeGeer et al. [21] revealed that bag dry aging will have no significant differences in *E. coli/coli* forms and lactic acid bacteria microbial growth than that of traditional dry aging. Shell loins aged in a bag will have about a 2 % yield advantage for combined weight loss over traditionally aged shell loins, whereas differences are minimal between strip loins regardless of the aging method. Gudjónsdóttir et al. [27] used electrospun chitosan fibres as a wrapping material for dry-aging beef showed improved results in terms of yield, reduction of microbial counts, yeasts and molds, and lighter appearance compared to traditional dry-aging.

Previous results suggest that the success and microbiological safety of dry aged beef is mostly dependent on controlled temperature and surface drying. Thus packaging in highly moisture-permeable bag may be positively impact on safety, quality and shelf stability of dry aged beef. However, Dikeman et al. [28] reported that neither dry nor special bag aging had advantages over wet aging. Sensory panel evaluation also showed no effect of aging method on myofibrillar tenderness, juiciness, connective tissue amount, overall tenderness or off flavor intensity [28].

## Eating quality of dry aged beef

### Meat flavor

The key effect of dry aging is the concentration of flavor that can only be described as “dry-aged beef”. Numerous workers have reported that eating dry aged beef is typically described as having a beefy, buttery rich, nutty, and/or earthy flavor profile. During the dry aging process, the juices are absorbed into the meat and chemical breakdown of protein and fat constituents occurs, resulting in a more intense nutty and beefy flavor [1, 4, 6, 21].

As beef ages, meat shows a significant alteration in the level of flavor precursors. The improvement of dry aged beef flavor may involve the reducing sugars, release of free amino acids, peptides and the breakdown of ribonucleotides to yield IMP, GMP, inosine, and hypoxanthine in meats during postmortem aging [29, 30]. Many of these changes are due to hydrolytic activity, although the activity of various hydrolases, such as the calcium-dependent, calpain proteinases implicated in fragmenting the muscle structure and the cathepsins implicated in the production of flavor peptides may play an important role in the temporal generation of flavor in meat during post-mortem aging [30]. Numerous workers have reported that the enzymes naturally in beef break down proteins to peptides and free amino acids during longer aging. The released aliphatic amino acids responsible for the sweet taste; while those containing a sulfur atom (Cys and Met) and Glu and Asp associated with the umami taste (MSG-like taste). Moreover carbohydrates broken down into sugars that give sweet taste, while fats and fat like membrane molecules degraded to aromatic fatty acids during aging. All of these breakdown products contribute to the intensely meaty, nutty and flavorful flavor of cooked dry aged steaks [12, 19, 31].

During cooking, flavor precursors also react with each other to form new molecules or volatile compounds that enrich the aroma further. It is a clever from chemical standpoint that dry aged beef could contain different flavor precursors or volatile flavor compounds than wet aged beef [10] while to our knowledge limited information about flavor components of proteolysis and lipolysis with the viewpoint of palatability of dry aged beef is available in the scientific literature. Most of the earlier research on the dry aging beef flavor has been concerned with the sensory traits affected by aging treatments (dry vs. wet) and dry aging bags [4, 8, 17, 21, 32].

Sensory analyses of dry and wet aged beef have revealed inconsistent results. Dry aged beef had a more beefy and brown roasted flavor than wet aged or unaged samples, while wet aged beef had more intense sour and metallic note and strong bloody/serumy flavor as determined by trained sensory panels [4, 6, 33]. Although dry aged samples had higher scores on some typical attributes compared to those wet aged e.g. umami, butter fried meat and nutty odor [21]. These results show that dry aging produces more flavorful beef than wet aging.

However, some studies have found that no differences in dry versus wet aged flavors evaluated by consumer panelists [8, 17, 33–36]. Periods of wet aging before or after dry aging were observed to exert little influence on development of dry aged flavor [6]. These studies indicate some consumers are more familiar with wet than dry aged flavors, but those who recognized or preferred the dry aged flavors were willing to pay more for that product [21].

Several studies have documented that dry aged beef flavor begins to develop after 14 days and intensifies thereafter, brown-roasted and nuttiness aromas were perceived for steaks dry-aged for 14 or 21 d. The longer it ages, the more intense and complex the flavors become, ranging from a subtle nuttiness to slight mushroom and umami flavors. After 45 days aging develops bold blue cheese notes [37]. According to Matsuishi et al. [38] dry aging for 20 days produced a sweet, milk-like aroma, which improved the flavor, but wet aging inhibited the development of desirable aged flavor and aroma. Moreover, Lepper-Blilie et al. [35] reported that overall aged flavor increases or concentrates as the days of aging increased. Days 42 and 49 had the highest aged flavor compared to days 14 and 21.

Numerous works have shown that undesirable flavors and aromas can be developed during aging due mainly to the effects of microbial growth, rancidity of the fat and adsorption of off-odors if present in the storage room [13].

### Tenderness

During dry aging process, the natural enzymes in the beef work to produce a more tender piece of meat than any you’ve experienced before [1]. Relative to this, Warren and Kastner [4] found that both wet and dry aging for 11 days resulted in tenderness scores that were significantly higher than the unaged controls. Consumer ratings for tenderness like increased for dry aged subprimals [6].

Length of aging was affecting WBSF values. The WBSF values for ribeyes and sirloins decreased with increased aging time [1]. Steaks dry aged for 14 days significantly improved in sensory tenderness compared to those dry aged 7 days [6] while, panelists found an improvement in tenderness when steaks reached 28 d of aging [35]. According to Smith [17] the significant decreases with a 17 % reduction in shear force from 14 to 35 days, showing that at least from an objective tenderness assessment standpoint, tenderness improvements were still occurring. The Gudjónsdóttir et al. [27] reported that muscle was partly denatured or degraded during dry aging. No significant difference was seen up to 14d of aging, while after 21d significantly more denaturation of the muscle of the traditionally dry aging beef.

However, some studies have shown improvements in tenderness with additional days of dry aging do not differ from wet aged counterparts obtained from same sources and handled in a similar manner [10]. Dry aging for 21 d produced steaks similar in tenderness to steaks dry aged for 14 d [6]. The continuing improvement in tenderness with aging by either method (vacuum or dry) beyond 14 d contrasts [6] with reports reviewed by researchers who found no significant improvements in tenderness after 11 or 14d [4, 33, 34].

Furthermore, Meat industry services Australia [2, 24] reported that processors need to be aware of the aging time, temperature required for development of optimum tenderness, and the pre- and post-slaughter conditions that influence aging. The degree of improvement in tenderness during aging is temperature dependent. The period of about 4 weeks at −0.5 °C would be required to achieve the same level of tenderness as 2 weeks at 5 °C. Whichever temperature is selected, the rate of improvement in tenderness is highest during the early stages of aging, and decreases with time [24].

Although ultimate pH of the meat can affect the degree of improvement in tenderness during aging and product selected for dry aging should come from carcasses with an ultimate pH 5.4 to 5.7. Muscles that have been cold shortened or heat shortened does not age as effectively as normally chilled meat [22, 24]. The maximum tenderness during beef aging differs depending on the muscles and the color of the lean. In general, dark beef age less easily compared to lighter colored meats. Also, the tenderness effect of beef aging is more apparent in meats from older animals in comparison to meat from younger animals [6, 35].

### Juiciness

Studies have found that there is an improvement in juiciness during dry aging. Steaks were significantly juicier after 21 d aging than after 14 d, which in turn gave steaks which were juicier than those for non-aged or aged 7 days [6]. Sensory results showed that many panelists preferred dry aged meat; dry-aged steaks were scored higher than wet aged steaks for juiciness. Dry aged meat was still juicy after cooking, but the juices are even more delicious than usual [21, 23].

This was attributed to a possible loss in water-holding capacity, resulting in more juices being released during chewing; The flavors and tissue itself becomes more concentrated by the loss of moisture during aging, actually increase the ratio of fat and that concentrated fat coats your palate. Also dry aged muscle fibers lose the ability to hold onto moisture, and so, when chew the meat; it is actually releasing more juice [6, 21, 23].

It is a well known fact that ultimate tenderness and improvement flavor of meat are dependent on the degree of alteration and weakening of myofibrillar structures and has been largely attribute to endogenous proteolytic enzymes [7]. Especially Ca^++^ dependent protease activity was determinant of tenderization resulting from postmortem aging [39]. But the greatest activity of enzyme is within the first 7 days of aging, and by 14 days the greatest gains in tenderness will have been achieved during aging [40]. However, what is happening after that? That is the peculiarity and where does the improvement in specific flavor come from during dry aging? Other novel proteolytic systems may contribute to post-mortem proteolysis and meat tenderization and the extent of which is yet to be fully examined [7]. This is a little less impressively documented between quality traits such as pH, collagen characteristics, tenderness, flavor and juiciness is at an optimum [12].

## Economic parameters

### Shrinkage

The interaction of aging treatment and period impacted on shrinkage (moisture loss) and total saleable yield percentages. Across all aging periods, dry aged ribeyes and strip loin sections had higher percentages of cooler shrink when compared to those that were wet-aged, with the 35 d dry-aged treatment having the highest [4, 21, 32]. Parrish et al. [16] showed that cooler shrink was evident in loins and ribs dry aged for 14 or 21 d, whereas products aged in vacuum packaged bags for the same time period resulted in little or no cooler shrink.

**Fig. 2:**
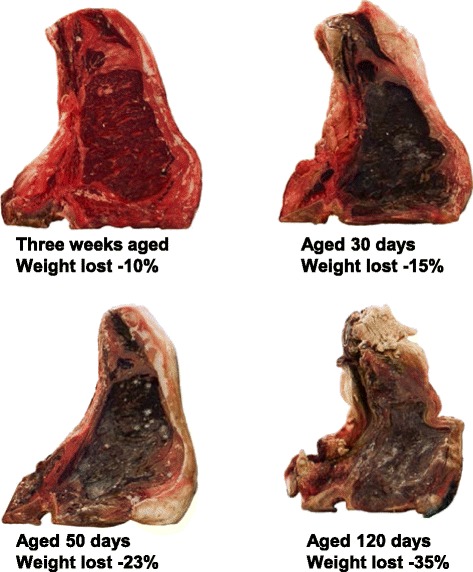
Weight loss of strip loins through dry aging process

Up to 5 % of the original weight of the carcass is lost during the dry aging process for 14 d aged beef. Overall shrinkage increased as the days of aging increased. Relative to this, “Pat LaFrieda” [41] explains how it works like shrinkage increase or intense beef flavor over the course of 120 days. At the 7 d aging, meat is still fairly bright, but it will darken as it ages and becomes drier. After 21d, steak loses 10 % of its weight through evaporation (Fig. 2). The water seeps out the front and the back of the meat, but the fat and bone on the sides of the steak make the sides waterproof. At the 30d aging, the steak has developed the flavor and texture qualities associated with dry-aged meat: It is very tender, with a flavor I can best describe as a mix of buttered popcorn and rare roast beef. At this point the steak has lost 15 % of its total weight, while steak has lost 23 % percent over 50 d. While at the 90 d aging, the white striations on the surface of the meat are good mold and also salt, which is extracted from the meat along with the water. The crust that develops around the meat protects it in the same way a rind does with cheese. The steak has lost 35 % of its original weight after 120 d aging. A steak aged this long has a very funky flavor and it is also very expensive, so it is for someone who really appreciates an intense beef flavor [41].

Moreover, the weight loss occasionally occurs at tremendous proportions depending on temperature air flow and relative humidity of the cooler room [12]. DeGeer et al. [21] reported that use of bone-in shell style loins would have economic advantages for weight and trim losses, over boneless product. Thus dry aging is usually done with primals on the bone. Because removing bone from loins accentuates greater moisture movement. However, additional trimming must be done by the consumer. In addition, weight loss affected by muscle type, dry aged shell loins lost more weight during aging compared with strip loins [21]. Carcasses or cuts with a thin external fat cover will lose more moisture than carcasses with a heavy fat covers, because of fat protect the meat from dehydration. Dry aged beef has been observed to require lower cooking losses and shorter cooking times than wet-aged [4].

### Fabrication yields

As dry-aging time was extended trimming time and amount of trim increased (Table 1) [6, 16, 17, 21, 32]. During beef aging for 7 to 21 days was a crust forms (removal of dried and discolored lean and fat) on the outside of the loin, very similar to the texture of beef jerky. This layer is trimmed away, leaving steaks that are superior in tenderness and flavor [21]. Smith et al. [17] found that dry aged short loins for all four different aging periods had significantly lower total saleable yield than their wet aged counterparts. The 28 d and 35 d dry aged ribeyes produced the lowest percentage of ribeye steaks (63.5 and 61.7 %) and had the highest percentage of waste trimmings (24.2 and 22.8 %), which would be expected with increased aging time [32]. Especially total saleable yields decreased from 72.2 to 63.5 % for ribs dry aged from 14 to 35 days. Steak yields were affected by cut type (generally bone-in ribeye > bone-in strip loin > boneless top sirloin [32] and USDA quality grade Choice generally less than Select [17, 21].

**Table 1:** The effect of aging treatment and aging time on retail cutting yields (%) of typical steaks

Item	Fabrication of cuts	Dry aged				Wet aged				References
		14d	21d	28d	35d	14d	21d	28d	35d	
Ribeye steaks	Ribeye	70.5^b^	66.7^c^	63.5^d^	61.7^d^	84.7^a^	83.7^a^	82.9^a^	83.3^a^	[32]
Beef for stew		0.3	2.2	0.7	1.6	1.8	2.4	2.2	3.6	
Lean trimmings		1.3	0.4	0.2	0.2	2	2.3	1.9	1.2	
Fat trim		2.5	1.7	-	-	5.1	5.7	5.5	5.6	
Waste trimmings		16.8^b^	17.3^b^	24.2^a^	22.8^a^	4.0^cd^	1.0^e^	5.4^c^	2.5^de^	
Cooler shrink		6.0^d^	7.6^c^	9.7^b^	11.7^a^	-	-	-	-	
Purge		0.8	0.8	0.5	0.6	0.2	0.4	0.5	0.6	
Total saleable yield		72.2^b^	69.3^c^	64.3^d^	63.5^d^	88.4^a^	88.4^a^	86.9^a^	88.1^a^	
Strip steaks	Strip loin	48.4	47	45	43.4	54.6	53.6	52.2	51	[18]
Vein steak		11.7	11.6	8.6	9.1	14	14.7	12.7	14.4	
Beef for stew		1.9	1.9	0.8	1.2	3.2	3.7	2	1.8	
Lean trimmings		0.1	0.4	1.4	0.9	0.6	0.9	2.8	2.2	
Fat trim		16.6^ab^	10.1^c^	6.3^c^	14.6^b^	20.3^a^	14.7^b^	19.1^a^	17.7^a^	
Waste trimmings		3.2^cd^	9.1^b^	16.0^a^	7.6^bc^	0.7^d^	4.9^cd^	4.6b^cd^	5.9^bc^	
Cooler shrink		8.2^c^	9.5^b^	11.2^a^	11.9^a^	1.0^d^	1.2^d^	0.8^d^	0.9^d^	
Purge		0.2	0.1	0.1	0.1	0.2	0.2	0.1	0.1	
Total saleable yield		62.1	60.8	55.9	54.7	72.4	72.9	72.9	69.8	
Top sirloin steaks	Top sirloins	57.3	54.1	50.4	48.7	75.8	75.6	67.9	70.2	[32]
Beef for stew		0.4^b^	-	1.2^b^	0.2b	0.7^b^	-	8.0^a^	-	
Lean trimmings		0.1	0.06	-	0.09	1.47	0.8	-	1.26	
Fat trim		11.3	10.5	11.8	13.3	15.9	15.9	20.5	18.5	
Waste trimmings		17.9^c^	21.0^ab^	23.6^a^	19.0^b^	-	-	-	-	
Cooler shrink		8.9^d^	11.2^c^	12.7^b^	15.0^a^	-	-	-	-	
Purge		0.43^e^	0.48^e^	0.3^e^	0.6d^e^	1.7^bc^	1.43^cd^	3.2^a^	2.4^ab^	
Total saleable yield		61.4^d^	56.5^e^	51.6^f^	52.0^f^	81.9^a^	82.5^a^	75.9^c^	78.6^c^	
Porterhouse steaks	Short loins	53.1	45.6	48.3	44.6	61.2	58.8	58.1	57.5	[17]
T-bone steaks		16	17.8	14.2	16.1	15.8	15.7	18.1	16.1	
Top loin steaks		5.2	6.4	7.0	7.1	6	4.7	5.8	7.2	
Beef for stew		1.2	1.5	1.4	1.1	1.8	3.3	2	2.5	
Lean trimmings		1.0	0.8	0.8	0.9	2.8	2.8	2.5	3.9	
Fat trim		4.1	5.7	4.8	4.4	4.6	7.1	6.4	4.4	
Waste trimmings		3.8	3.5	4.2	4.5	-	-	-	-	
Cooler shrink		5.4^b^	6.0^b^	6.1^b^	8.5^a^	-	-	-	-	
Purge		0.1^d^	0.5^cd^	0.6^bc^	0.3^cd^	1.1^a^	0.6^bc^	1.0^ab^	1.1^a^	
Total saleable yield		76.5^c^	72.1^d^	71.6^de^	69.8^e^	87.7^a^	85.3^b^	86.6^ab^	87.1^ab^	

^a-f^, means within row with different superscripts are significantly different

Studies done by Smith et al. [17] have showed a significant increase in the time required to process dry vs. wet-aged short loins into steaks and other saleable products (dry-aged: 331.6 sec per shortloin; wet-aged: 243.1 s per shortloin). Much of this increased processing time was due to the removal of dried and discolored lean and fat (referred to as “crust” in the industry) from the dry-aged compared to the wet-aged shortloins. There was a trend towards increasing processing times with increased aging times but these differences were less evident compared to those found between the dry-aged and wet-aged subprimals. Laster et al. [32] also reported significant increases in time required to cut bone-in ribeyes, bone-in striploins, and top sirloin butts for dry-aged versus wet-aged products.

### Cost/pricing

Dry aging of beef is a costly procedure because of decreased yields due to greater weight, trim losses and time consuming processing compared with wet aging. Moreover for aging to properly improve the quality of a cut of meat, it should contain substantial marbling. This means that there is fat evenly distributed throughout the meat. Only the highest grades have this kind of marbling and make aging worthwhile [13].

The aging treatment has a significant impact on total cutting time. Smith et al. [17] found a greater time associated with processing dry-aged short loins was directly related to the removal of the “crust” before short loin fabrication. Total cutting time (sec) of per short loins stratified by aging treatment (dry vs. wet aging) was 331.6 s and 234.1 s, respectively. Aging period also significantly affected total cutting time with short loins aged for 28 and 35 d having the highest total cutting times [17].

Smith et al*.* [17] calculated that retail prices of dry-aged steaks from short loins would need to be up to 19 % higher to return the same net sales value and margin as obtained from wet-aged short loins. Mostly dry-aged beef usually cost about 25 % more than wet aged beef.

Results of previous study showed that sub primal cuts slowly dehydrating and losing water during dry aging, concentrates the flavor but also loses about 5–25 % of its starting weight. This is predominantly the reason that this type of high quality product costs more than the non-dry aged product. However, it will reward producers with the most tender and truly naturally flavorful beef.

## Conclusion

In conclusion, dry aging is a process to produce unique flavored, value added beef. However, dry aging is a costly endeavor due to aging conditions needed for proper dry aging to occur to achieve proper palatability. This process also requires the highest grades beef with necessary marbling. However, there is a niche market of discerning consumers who are willing to pay for this premium product. On the other hand, there is no much accessible information on interaction between aging parameters and microbiology on the quality of dry aged beef and consequently beef palatability. This area of research is less explored, and many questions related to this area remain unanswered. Considering the dramatic raise of demand dry aged beef product, studies targeting to this processing need to be conducted, as the guidelines and recommendations on aging conditions that should be help companies or retailers who interested in producing a dry aged beef seems more necessary than ever.
