# Erratum to: Dry aging of beef; Review

**DOI:** 10.1186/s40781-016-0109-1

**Published:** 2016-07-24

**Authors:** Dashmaa Dashdorj, Vinay Kumar Tripathi, Soohyun Cho, Younghoon Kim, Inho Hwang

**Affiliations:** Department of Animal Science and Biotechnology, Chonbuk National University, Jeonju, 561-756 Republic of Korea; Department of Livestock Production, Mongolian University of Life Sciences, Ulaanbaatar, 17026 Mongolia; Animal Products Research and Development Division, National Institute of Animal Science, RDA, Wanju, Republic of Korea

## Erratum

After publication of the original article [1], the publisher was notified by the author that permission to reproduce the three images comprising Fig. 1, “The refrigerated coolers for dry beef”, had not been obtained. As a result, Fig. 1 has been removed from the original article and Fig. 2 has been renumbered to Fig. 1.
